# Meiosis in bulbous flower species *Lycoris*: dances underground

**DOI:** 10.3389/fpls.2025.1691599

**Published:** 2026-01-02

**Authors:** Ziming Ren, Jingru Wang, Nan Huang, Huiqi Fu, Bing Liu, Yiping Xia

**Affiliations:** 1 Laboratory for Regulation of Key Traits and Germplasm Innovation in Bulbous and Perennial Ornamentals, School of Civil Engineering and Architecture, Zhejiang Sci-Tech University, Hangzhou, China; 2 Arameiosis Lab, South-Central Minzu University, Wuhan, China; 3 Genomics and Genetic Engineering Laboratory of Ornamental Plants, Department of Horticulture, College of Agriculture and Biotechnology, Zhejiang University, Hangzhou, China

**Keywords:** meiosis, allotriploid, diploid gamete, chromosome, homologous recombination, *Lycoris*

## Abstract

*Lycoris*, a perennial bulbous flower species, is valued for ornamental features and abundant medicinal ingredients. The reproductive development features of *Lycoris*, especially meiosis, remain largely uncharacterized, which hinders its breeding programs. However, the references for investigating meiosis in *Lycoris* are limited at present. In addition, a special reproductive trait of *Lycoris* that typically differs from other species is that its meiosis occurs in bulbs underground, which increases the difficulties in cytological dissection of sexual cells. In this study, we analyzed meiotic chromosome behaviors in two diploid *Lycoris* species (*L.* sp*rengeri* and *L. aurea*) and two naturally-derived allotriploid varieties (*L. chunxiaoensi* and *L. hubeiensis*). The correlation of anther size and the stage of meiosis was determined, which revealed differences between diploid species. Diploid *Lycoris* showed defects in chromosome segregation, indicating that meiosis in *Lycoris* is unstable. Meiotic restitution, which defines non-reductional meiosis events, was observed in both diploid species, implying a potential to yield unreduced gametes and thus may explain the natural derivation of polyploids. Immunolocalization of the recombinase HEI10 revealed that *L.* sp*rengeri* and *L. aurea* have similar class-I type crossover rates. Moreover, we showed that allotriploid *Lycoris* exhibit severely disrupted chromosome pairing and bivalent formation, the levels of which varied between varieties. These meiotic defects leaded to aneuploid meiotic products and sterility. Taken together, this study provides a cytological reference and insights into meiosis features in *Lycoris*, which paves a rode for further studies on reproductive biology and genetics in this special bulbous flower species.

## Introduction

Meiosis is a specialized type of cell division, during which chromosomal DNA is replicated once while chromosomes segregate twice at meiosis I and II leading to production of daughter cells with a halved chromosome number. At prophase I (P I), chromosomes in sexual cells, which, in flowering plants, are pollen mother cells (PMCs) and megaspore mother cells (MMCs), undergo a series of complex movement and conformational alterations (Cromer et al., 2024). At this stage, homologous chromosomes (homologs) undergo meiotic recombination (MR), which creates genetic diversity that drives genome evolution and enables environmental adaption, and maintains genome stability by facilitating faithful chromosome separation (Zickler and Kleckner, 2023). MR is initiated by the formation of double-strand breaks (DSBs) that are generated by SPO11, a relative of archaeal topoisomerase VI, together with other function-related proteins in the DSB-catalyzing complex (Vrielynck et al., 2021). DSBs are repaired through the recombination pathway mediated by the recombinases RAD51 and DMC1 (Da Ines et al., 2013; Kurzbauer et al., 2012). Synchronously, chromosome axis is built to facilitate homolog pairing and recombination (Chambon et al., 2018; Ferdous et al., 2012). Paired homologs further synapse based on the assembly of the synaptonemal complex, which plays an important role in regulating normal crossover (CO) rate (France et al., 2021; Lake and Hawley, 2021; Yang et al., 2022). COs between homologs lead to formation of bivalents (in a diploid organism). A mechanism termed ‘CO assurance’ ensures that at least one CO is formed between a homolog pair (Jones and Franklin, 2006), which is crucial for balanced chromosome separation and thus viable gamete production (Wang and Copenhaver, 2018). Homologs and sister chromatids segregate at anaphase I and II, respectively, which rely on the assembly and organization of microtubular cytoskeleton (Zamariola et al., 2014).

Defects in meiosis, e.g., impaired meiotic recombination, aberrant spindle and phragmoplast assembly or organization, and untimely disjoining of sister chromatids, may result in irregular nuclei distribution, which consequently leads to production of aneuploid gametes and associated reduced or impaired fertility (Mercier et al., 2015). On the other hand, lesions in spindle and phragmoplast orientation, omission of meiotic cell cycle and incomplete meiotic cytokinesis may trigger meiotic restitution, a phenomenon that defines non-reductional meiosis events, which ultimately generates diploid and/or polyploid gametes and thus polyploid offspring (Brownfield and Kohler, 2011; Mason and Pires, 2015).

Most angiosperms have experienced whole genome duplication (WGD), which plays an important role in their genome evolution, speciation, and environmental adaption (Ren et al., 2018; Soltis et al., 2015; Van de Peer et al., 2020). An accepted notion is that formation and fusing of unreduced gametes contribute to the origin of WGD in flowering plants (Ramsey and Schemske, 1998). In polyploids, especially those having not experienced meiosis adaption, increased sets of chromosomes challenge homolog pairing and recombination (Lloyd and Bomblies, 2016), which attenuates obligate CO formation leading to disordered chromosome segregation and reduced fertility (Fu et al., 2022; Lloyd and Bomblies, 2016; Morgan et al., 2021).

*Lycoris* Herb., an important bulbous species belonging to the Amaryllidaceae family with strong growth adaptability and stress resistance, is highly valued for its ornamental features and abundant medicinal ingredients (Ren et al., 2017, 2022). Because of a long growth cycle of *Lycoris*, which generally goes through four- to six-year vegetative development before flowering, breeding of *Lycoris* is challenging and thus vegetative propagation is more preferentially conducted at present. However, this breeding strategy cannot fully utilize the potential of genetic diversity in the *Lycoris* genomes. Natural hybridization is a common route to speciation in *Lycoris* (Liu et al., 2019), which generates natural hybrids with different physiological traits and complex genetic compositions (Jiang et al., 2020; Li et al., 2020; Liu et al., 2019; Quan et al., 2024; Zhang et al., 2025). At present, there are twenty-seven legitimate diploid *Lycoris* species with only nine of them being considered original fertile species, including *L. chinensis*, *L.* sp*rengeri*, *L. radiata*, *L. longituba*, *L. aurea*, *L. traubii*, *L. sanguinea*, *L. wulingensis*, and *L. tsinlingensis* (Zhang et al., 2022). However, a lack of genomic information and tools for evaluating genetic diversity hinders the efficient selection of parental inbreds for hybridization. Moreover, the reproductive development features in *Lycoris*, especially meiosis, remain largely unclear. At present, the references for studying reproductive biology and genetic in *Lycoris* are limited. A special reproductive trait of *Lycoris* that typically differs with other plant species is that its meiosis occurs in bulbs at a developmental stage underground. This physiological feature increases the difficulties in dissecting reproductive organs including sexual mother cells in *Lycoris*. On the other hand, an extensive occurrence of interspecific hybridization between diploid *Lycoris* with overlapped wild habitat leads to generation of allotriploid *Lycoris*, such as *L. hupehensis* (Meng et al., 2018) and *L. chunxiaoensis* (Li et al., 2022), which are considered having a strong environmental adaptability and high horticultural utilization values. Nevertheless, most *Lycoris* hybrids including polyploids have poor seed setting and thus are vegetatively propagated via bulb scale cuttings, which generates substantial clonal populations in natural habitats (Meng et al., 2018; Zhang et al., 2022). The mechanisms underpinning the low fertility in *Lycoris* and natural derivation of polyploids remains elusive.

In this study, we dissected chromosome behaviors during male meiosis in two diploid *Lycoris* species and two naturally-derived allotriploid varieties. We report here that diploid *Lycoris* have defects in meiotic chromosome segregation and undergo meiotic restitution, which reveals meiotic instability in *Lycoris*. Allotriploid *Lycoris* show defects in homologous chromosome pairing and CO formation, the levels of which differ between varieties. This study provides a cytological reference and insights into meiosis features and thus paves a road for further studies on the reproductive biology and genetics in *Lycoris*.

## Results

### Correlation of meiosis progression with the anther size varies between diploid *Lycoris* species

**Figure 1:**
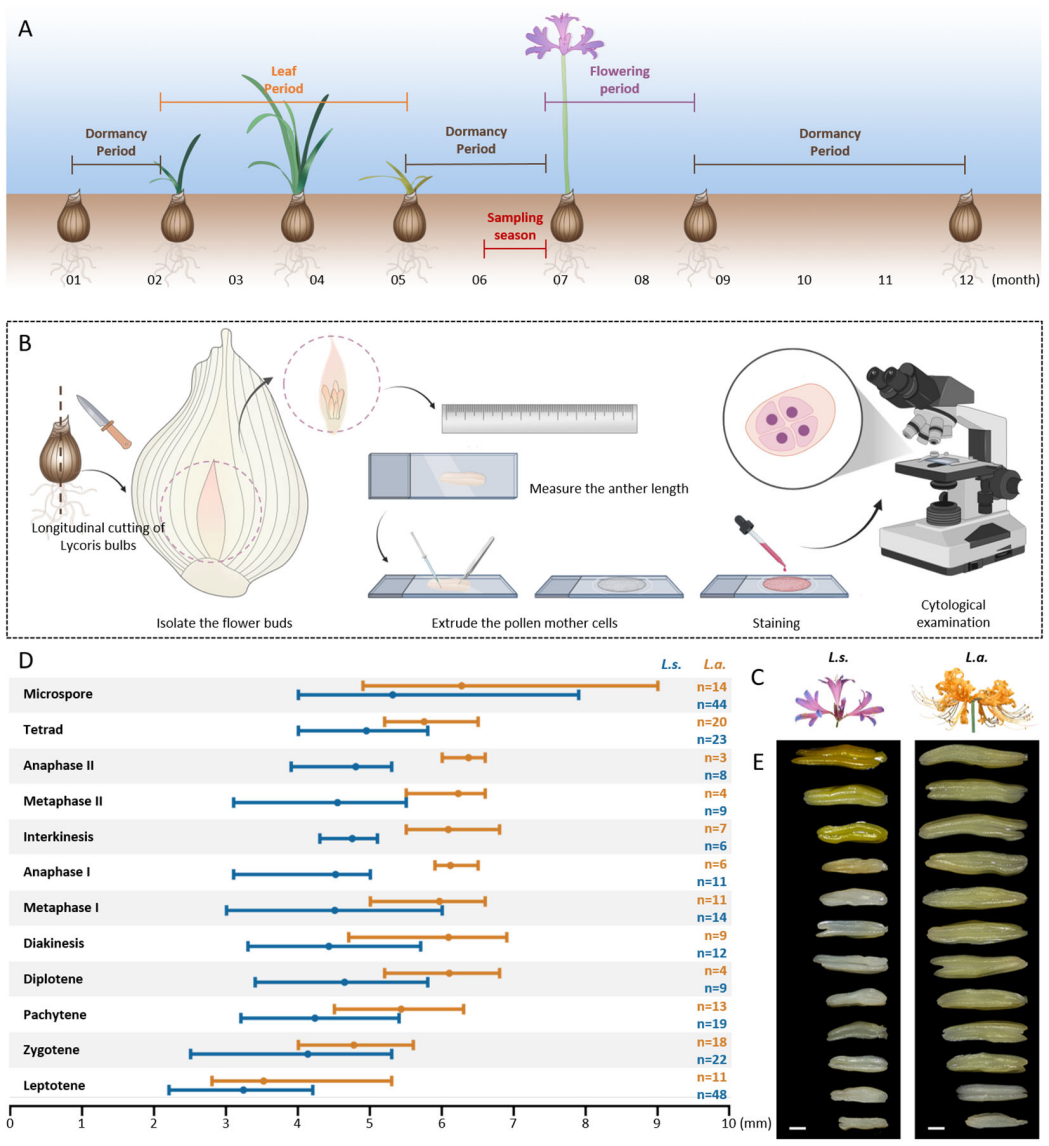
*L. aurea* shows larger sizes of the anthers harboring pollen mother cells (PMCs) undergoing meiosis than *L.* sp*rengeri*. **(A)** Illustration of the development progression of *Lycoris* in a single year. **(B)** Illustration of the method for staging meiosis and measurement of the anther size. **(C)** Booming flowers showing different colors in *L.* sp*rengeri*. and *L. aurea*. **(D)** Graph showing the sizes of the anthers harboring PMCs at the corresponding meiosis stages in *L.* sp*rengeri*. and *L. aurea*. n indicates the number of the analyzed anthers. **(E)** Anthers at different development stages in *L.* sp*rengeri*. and *L. aurea*. The panels **(A, B)** were created in BioRender.

*Lycoris* exhibits a unique developmental character which is that its leaves and flowers emerge asynchronously. Taking the spring-leafing *L.* sp*rengeri* (*L.s.*) as an example, its leaves emerge approximately from February while wither in May, thereafter entering into an aboveground dormancy period prior to reproductive development (**Figure 1A**). Generally, *Lycoris* undergoes meiosis in June and flower from July to September, and interestingly, its meiosis and gametogenesis processes occur in the anthers wrapped in bulbs (**Figure 1A**). This special reproductive developmental feature of *Lycoris* raises the difficulties of sampling meiotic flower buds and intuitively staging the anthers via cytological tools as in other species (**Figures 1A, B**). Therefore, we examined the correlation of meiosis stages and the size of anthers in *Lycoris*. We analyzed meiosis stage of the pollen mother cells (PMCs) and measured the length of the corresponding anthers in which PMCs were isolated in *L.s.* and *L. aurea* (*L.a.*), two native diploid *Lycoris* species with differences in multiple development traits, especially the flower color (**Figures 1B, C**) (Li et al., 2020; Ren et al., 2017, 2022). In both *L.s.* and *L.a.*, PMCs at different stages were observed in every single anther, indicating an unsynchronized development of PMCs in *Lycoris*. Generally, *L.a.* showed larger sizes of the anthers (on average 3.5 to 6.3 cm) that contained PMCs from early meiosis to microspore stages than *L.s.* (on average 3.0 to 5.2 cm), especially in meiosis II (**Figures 1D, E**).

### Diploid *Lycoris* species show meiotic instability and undergo meiotic restitution

**Figure 2:**
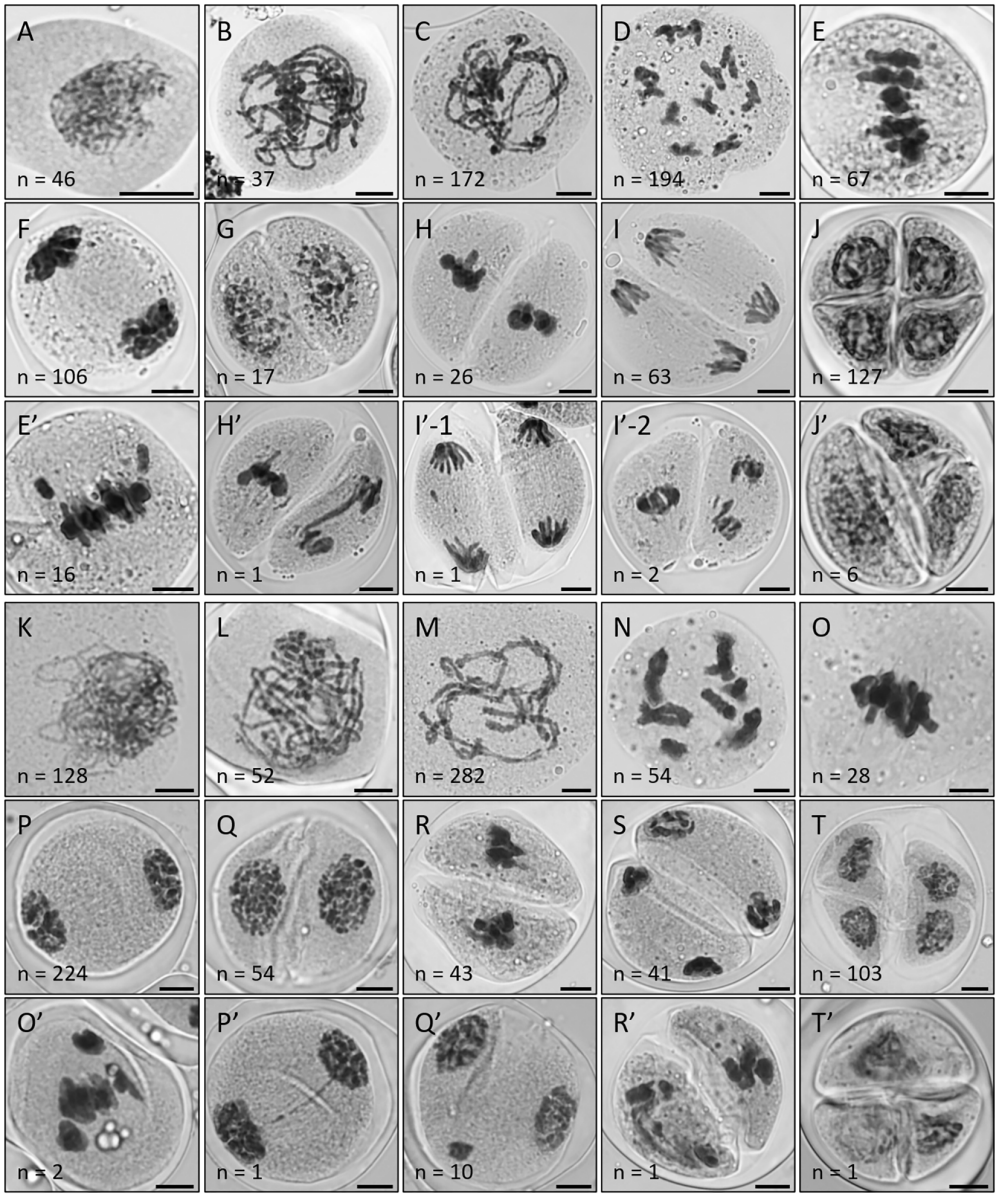
Diploid *Lycoris* show male meiotic instability and meiotic restitution. **(A-T’)**, Orcein staining of PMCs at zygotene **(A, K)**, pachytene **(B, L)**, diplotene **(C, M)**, diakinesis **(D, N)**, M I **(E, E’, O, O’)**, late A I **(F, P, P’)**, interkinesis **(G, Q, Q’)**, M II **(H, H’, R, R’)**, T II **(I, I’-1, I’-2, S)** and tetrad **(J, J’, T, T’)** stages in *L.* sp*rengeri***(A-J’)** and *L. aurea***(K-T’)** showing normal **(A-J, K-T)** and abnormal **(E’-J’, O’-T’)** chromosome behaviors. Scale bars = 10 μm.

To explore the general features of meiotic chromosomes in *Lycoris*, we stained PMCs in *L.s.* and *L.a.* with orcein, which exhibited an average diameter of approximately 57.5 and 63.3 µm, respectively, with *L.a.* showing a larger size of PMCs than *L.s.*, especially during meiosis I (**Supplementary Figures S1A, B**). The large sizes of meiocytes in *L.s.* and *L.a.* roughly provided a clear visualization of the chromosome configuration during male meiosis. Both *L.s.* and *L.a.* showed normal chromosome behaviors and configurations during prophase I (P I) (**Figures 2A–D**, *L.s.*; **K-N**, *L.a.*). Specifically, at pachytene, homologs were fully paired and juxtaposed indicating successful synapsis (**Figures 2B, L**). All the observed PMCs at diakinesis in *L.s.* and *L.a.* produced eleven and seven pairs of bivalents, respectively (**Figures 2D, N**). At metaphase I (M I), homologs were aligned at the cell plates by the bipolar pulling force from a spindle (**Figures 2E, O**), which segregated to the opposite cell poles at anaphase I (A I) (**Figures 2F, P**). After a short period of decondensation at interkinesis, homologs condensed again and were aligned at the cell plates at metaphase II (M II) (**Figures 2G, H, Q, R**). By the pulling force from two spindles, sister chromatids separated at telophase II (T II), which developed into four haploid nuclei with each being surrounded by a cell wall in a tetrad that manifested completion of male meiosis (**Figures 2I, J, S, T**).

In both *L.s.* and *L.a.*, PMCs at M I with abnormal chromosome behaviors including defective alignment and distribution were observed, and the rate of these defects in *L.s.* (19.3%, n = 83) was higher than that in *L.a.* (6.7%, n = 30) (**Figures 2E’, O’**). In addition, asynchronous and lagged segregation of sister chromatids were occasionally visualized at A I and II (**Figures 2H’, I’-2; P’, R’**). Notably, around 4.3% (n = 234) PMCs at A I in *L.a.* displayed an unbalanced distribution of chromosomes, which will likely lead to aneuploid nuclei at the end of meiosis. These observations indicated that chromosome arrangements and segregation during male meiosis in diploid *Lycoris* is unstable. Remarkably, PMCs at the tetrad stage showing a triad-like configuration that consisted of one diploid microspore and two haploid microspores occurred at 4.5% (n = 133) and 1.0% (n = 104) in *L.s.* and *L.a.*, respectively (**Figures 2J’, T’**). These triads suggested an occurrence of meiotic restitution in diploid *Lycoris*, which hints a potential to generate unreduced microspores.

### Diploid *Lycoris* show defects in meiotic chromosome segregation and distribution

**Figure 3:**
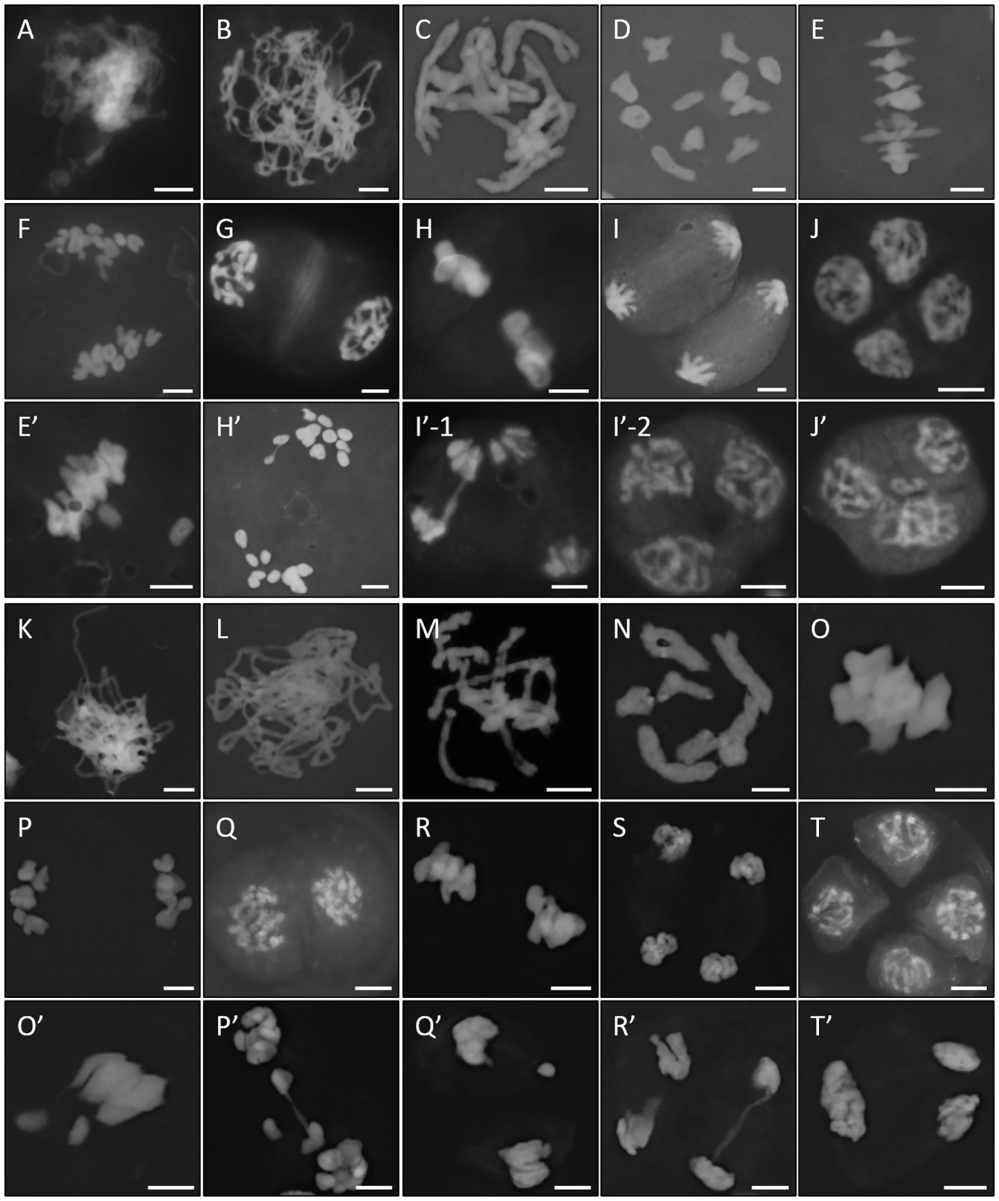
Diploid *Lycoris* show defects in meiotic chromosome separation and distribution. **(A-T)**’, DAPI staining of chromosomes at zygotene **(A, K)**, pachytene **(B, L)**, diplotene **(C, M)**, diakinesis **(D, N)**, M I **(E, E’, O, O’)**, A I **(F, H’, P, P’)**, interkinesis **(G, Q)**, M II **(H, R, Q’)**, T II **(I, I’-1, S, R’, T’)** and tetrad **(J, I’-2, J’, T)** stages in *L.* sp*rengeri***(A-J’)** and *L. aurea***(K-T’)** showing normal **(A-J, K-T)** and abnormal **(E’-J’, O’-T’)** behaviors. Scale bars = 10 μm.

To further characterize the chromosome features during meiosis in diploid *Lycoris*, we prepared chromosome spreads in *L.s.* and *L.a.* by performing 4’,6-diamidino-2-phenylindole (DAPI) staining, which has been widely used to precisely dissect meiotic chromosome structures in many plants. In both diploid *Lycoris* species, meiotic chromosomes displayed regular homolog pairing and bivalent formation, which indicated normal meiotic recombination (**Figures 3A–D, K–N**). However, scattered chromosomes at M I (**Figures 3E, O**, normal; **E’, O’**, abnormal) and irregular chromosome association and distribution were visualized from A I to T II (**Figures 3F–I, P–S**, normal; **H’, I’-1, P’, R’**, abnormal). At tetrad stage, PMCs with an irregular number of nuclei or mini-nucleus were observed in *L.s.* (**Figures 3J, I’-2, J’**), and triad-like PMCs were found in *L.a.* (**Figures 3T, T’**). These data confirmed that diploid *Lycoris* have defects in chromosome segregation and distribution during male meiosis. Since no obvious defects in chromosome pairing and bivalent formation were detected in diploid *Lycoris*, the lesions in chromosome segregation and distribution likely occur after meiotic recombination.

### *L.* sp*rengeri* and *L. aurea* show similar rates of type-I crossover

**Figure 4:**
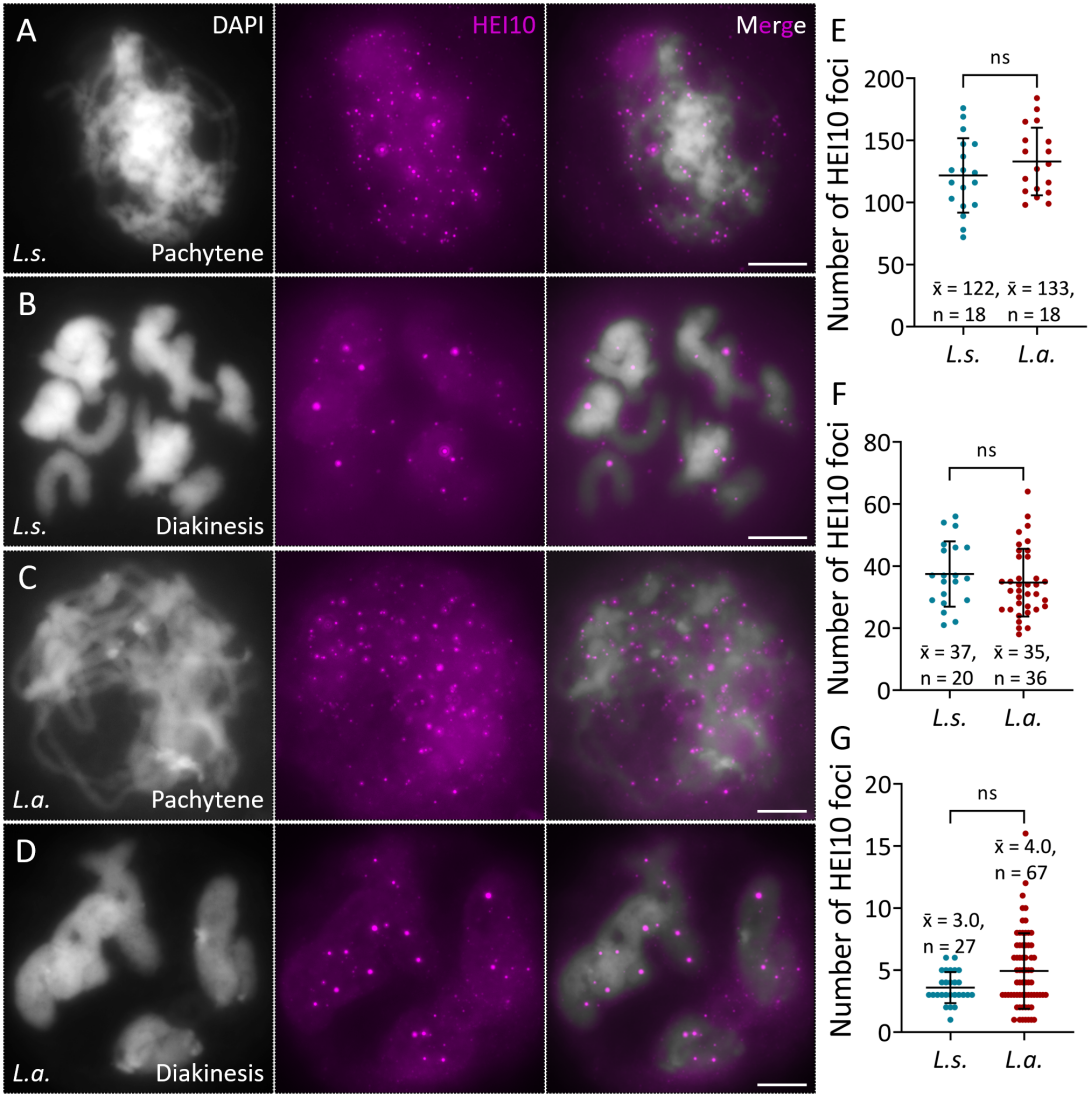
*L.* sp*rengeri* and *L. aurea* show similar rates of type-I CO. **(A-D)**, Immunolocalization of HEI10 protein on pachytene **(A, C)** and diakinesis **(B, D)** chromosomes in *L.* sp*rengeri***(A, B)** and *L. aurea***(C, D)**. White, DAPI; pink, HEI10. Scale bars = 10 μm. **(E, F)**, Graphs showing the number of HEI10 foci on pachytene **(E)** and diakinesis **(F)** chromosomes in *L.* sp*rengeri* and *L. aurea*. **(G)** Graph showing the numbers of HEI10 foci per bivalent in *L.* sp*rengeri* and *L. aurea*. The significance levels were determined based on unpaired *t* tests; the average values are indicated; n indicates the number of PMCs **(E, F)** or bivalents **(G)**; ns indicates *P* > 0.05.

We compared the crossover (CO) rates in *L.s.* and *L.a.* by scoring the number of the type-I class CO recombinase HEI10 foci on pachytene and diakinesis chromosomes. Immunolocalization of HEI10 was performed using an antibody generated using a peptide of HEI10 protein in Arabidopsis as the antigen (Fu et al., 2022). At pachytene stage, a large number of HEI10 foci was detected in both species, and there was no significant difference in between (120.0 vs. 129.0) (**Figures 4A, C, E**, P > 0.05). At diakinesis, the average numbers of HEI10 foci per PMC in *L.s.* and *L.a.* decreased to 36.5 and 33.0, respectively, also without a significant difference in between (**Figures 4B, D, F**, P > 0.05). Considering the difference in the number of homolog pair between *L.s.* and *L.a.*, we speculated that they may have a difference in the average number of CO per bivalent. To this end, we scored and compared the number of HEI10 foci on a single bivalent between these two species, which, however, revealed no significant difference (**Figure 4G**; *P* > 0.05). While, it was noted that *L.a.* showed a larger variation in the number of HEI10 foci between bivalents (**Figure 4G**). These data suggested that *L.s.* and *L.a.* have the same level of type-I CO.

### Naturally-derived allotriploid *Lycoris* shows severe meiosis defects that vary between varieties

**Figure 5:**
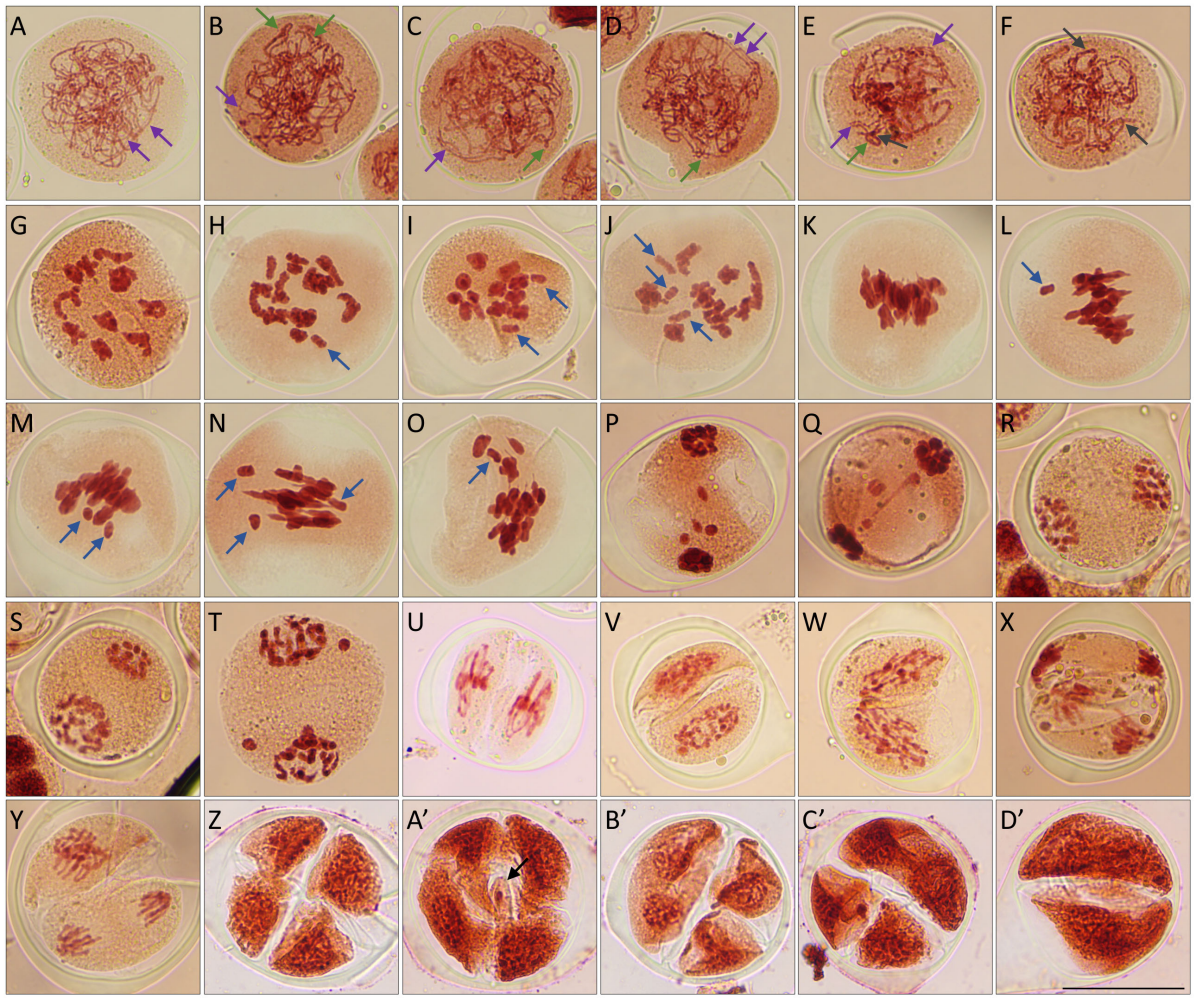
Allotriploid *Lycoris* shows severe defects in multiple meiosis processes. **(A-D’)**, Orcein staining of PMCs in *L. chunxiaoensis* at pachytene **(A-F)**, diakinesis **(G-J)**, M I **(K-N)**, A I **(O-Q)**, interkinesis **(R-T)**, M II **(U)**, A II **(V, W)**, T II **(X, Y)** and tetrad **(Z-D’)** stages. The purple arrows indicate unpaired chromosome regions; green arrows indicate chromosome loops; gray arrows indicate regions showing irregular coarsening; blue arrows indicate univalents; black arrow indicates mini-nucleus. Scale bar = 50 μm.

**Figure 6:**
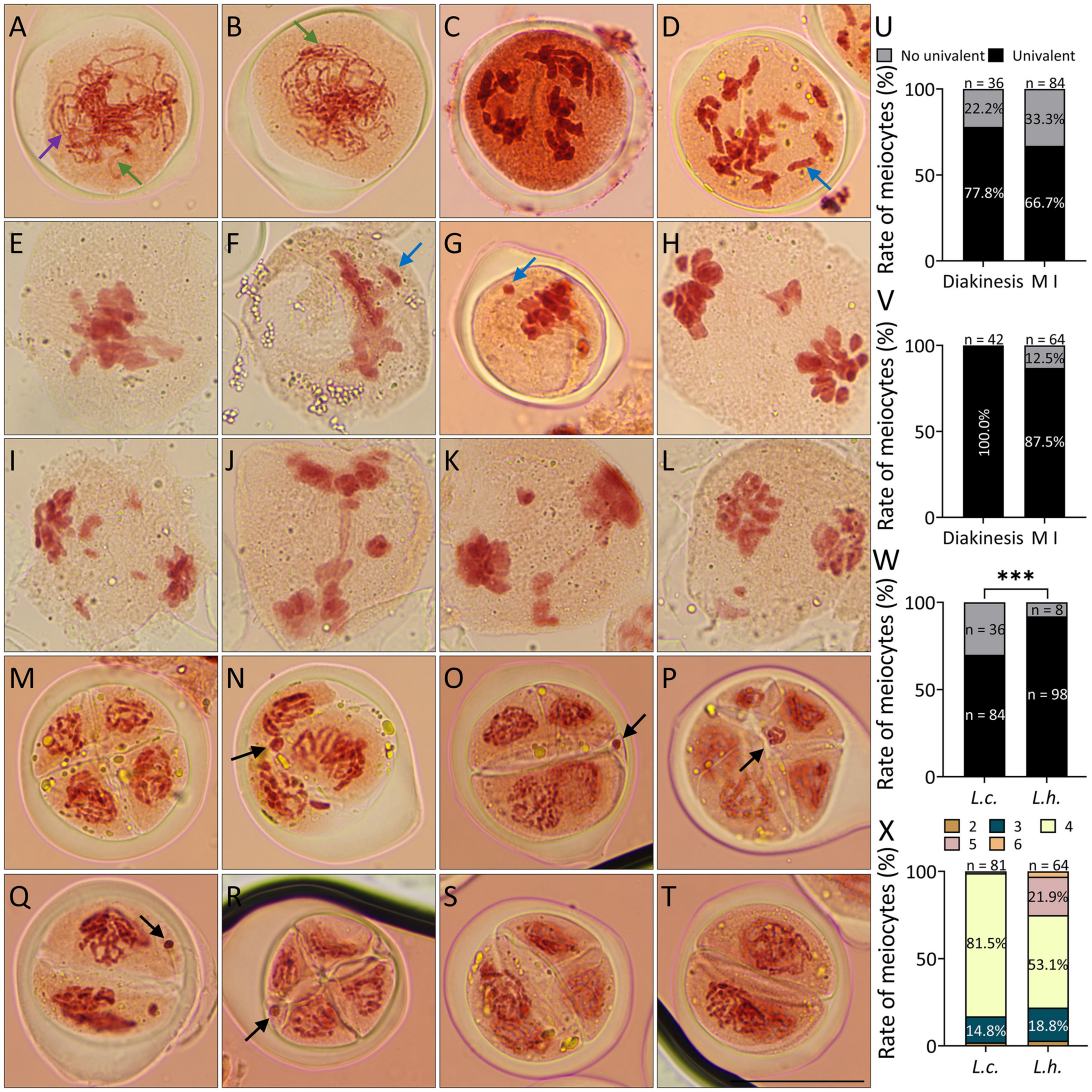
Allotriploid *Lycoris* varieties show different levels of meiosis instability. **(A-T)**, Orcein staining of PMCs in *L. hupehensis* at pachytene **(A, B)**, diakinesis **(C, D)**, M I **(E-G)**, A I **(H-J)**, interkinesis **(K, L)** and tetrad **(M-T)** stages. The purple arrows indicate unpaired regions; green arrows indicate chromosome loops; blue arrows indicate univalents; black arrow indicates mini-nucleus. Scale bar = 50 μm. **(U, V)**, Graphs showing the rates of PMCs at diakinesis and M I with univalents in *L. chunxiaoensis***(U)** and *L. hupehensis***(V)**. **(W)**, Graph showing the rates of PMCs at both diakinesis and M I stages with univalents in *L. chunxiaoensis* and *L. hupehensis*. **(X)**, Graph showing the rates of PMCs at tetrad stage showing different numbers of nuclei. The significance level was determined based on chi-squared test; *** indicates *P* < 0.001; n indicates the number of PMCs; rates indicate the frequencies of the corresponding phenotypes.

Natural hybridization is a common route to speciation in *Lycoris*, which generates hybrids including allotriploid species with complex genome compositions (Liu et al., 2019, 2015; Shu et al., 2025; Zhang et al., 2025). We further analyzed meiotic chromosomes in two naturally-derived allotriploid *Lycoris* varieties, *L. chunxiaoensis* (*L.c.*) and *L. hupehensis* (*L.h.*). At the pachytene-like stage, when chromosomes looked obviously thicker and more condensed and showed a juxtaposed configuration, PMCs in both varieties exhibiting unalignment with partner chromosomes were frequently observed, which indicated defects in homolog pairing (**Figures 5A–E**; **6A**; purple arrow). In addition, we also observed loop-like chromosome structures in the triploid *Lycoris* varieties (**Figuress 5B–E**; **6A, B**; green arrows), which suggested defective chromosome pairing and/or other irregular chromosome associations. Moreover, in *L.c.* we observed that there were some regions thicker than other chromosome regions (**Figure 5F**; black arrows), which hinted an irregular homolog or non-homolog association (Morgan et al., 2017). Notably, in *L.c.*, 77.8% and 66.7% PMCs at diakinesis and M I stages, respectively, showed univalents, and the values were 100.0% and 87.5%, respectively, in *L.h.* (**Figures 5G–O**; **6C–G**, blue arrows; **6U** and **6V**). Significance analysis revealed that *L.h.* produced a higher fraction of PMCs with univalents at diakinesis and M I stages than *L.c.* (**Figure 6W**, *P* < 0.001). These findings demonstrated that crossover formation is severely interfered in allotriploid *Lycoris*. At the end of meiosis I, *L.c.* and *L.h.* displayed irregular homolog segregation and distribution (**Figures 5P–T**; **6H–L**), which leaded to disordered chromosome distribution and thus production of polyads instead of tetrads at the end of meiosis II (**Figures 5Z, A’**; **Figures 6M**, normal tetrad; **6N–R**, PMCs with mini-nucleus). Interestingly, we observed triangle-like orientation and failed separation of sister chromatids at A II and T II in *L.c.* (**Figures 5U, V**, normal A II; **5W**, triangle-like; **5X**, normal T II; **5Y**, unseparated sister chromatids). Consequently, triad- and dyad-like PMCs were formed at the tetrad stage (**Figures 5B’, C’**, normal tetrad; **5U**, triads; **5D**’, dyad). Similar cellular defects were visualized in *L.h.* (**Figures 6S**, triad; **6T**, dyad). The fraction of PMCs at the tetrad stage with an irregular number of nuclei in *L.h.* was higher than that in *L.c.* (**Figure 6X**). The lesions in chromosome distribution in allotriploid *Lycoris* could result from the defects in chromosome pairing and CO formation, and also partially from triploidy-induced unbalanced chromosome segregation. These findings revealed that allotriploid *Lycoris* have severely disrupted meiosis, which will likely induce sterility. In line with this notion, our fluorescein diacetate (FDA) staining assay revealed that compared with diploid *Lycoris* species, allotriploid *Lycoris* had a dramatically reduced pollen viability together with an impaired seed setting (**Supplementary Figures S2**-**4**).

## Discussion

Reproductive development features of *Lycoris* remains largely uncharacterized, which hinders its breeding efficiency. *Lycoris* has evolved a special reproductive development trait typically different from other plant species including other bulbous flower species, which is that meiosis occurs in bulbs, additionally, underground. This feature raises difficulties for studying meiosis in *Lycoris* using cytological, molecular, microscopic and omics approaches, which rely on precise staging and/or isolation of the anthers and/or meiocytes (Chen et al., 2010; Huang et al., 2020, 2019; Prusicki et al., 2019; Wang et al., 2014). In this study, we determined the correlation of the anther size and the stage of meiosis in diploid *Lycoris*, which showed variation between species. This work provides a reference for the precise staging of the anthers in study of *Lycoris* meiosis, and, may benefit studies that involve stresses (Kacprzyk et al., 2025). We dissected meiotic chromosome behaviors by applying different staining methods in two diploid species and two allotriploid varieties, which provides a methodological reference for studying meiosis and sheds light on features of meiotic homologous chromosome pairing, recombination and chromosome segregation in *Lycoris*. We propose that *Lycoris* can be used as a model for studying meiosis in bulb flower species because of its special physiological traits, large size of meiocytes, and the ease that meiotic stages can be cytologically defined as well as its abundant resources (Deng Chuan-Liang et al., 2006; Gao et al., 2014; Jiang et al., 2020; Li et al., 2020; Zhang et al., 2019).

Both *L.s.* and *L.a.* produce irregular meiotic products at tetrad stage, which indicate that male meiosis in diploid *Lycoris* species is unstable. The diploid *Lycoris* did not show defects in homolog pairing and bivalent formation, the observed irregular alignment of chromosomes at M I thus does not likely result from lesions in crossover formation, but may be caused by defects in chromosome dynamics at later meiosis stages, e.g., spindle-mediated chromosome segregation. Irregularities of meiotic chromosome separation and distribution have been observed in petunia (Petunia X hybrida) (Fu et al., 2024), which are considered to be owing to an interfered spindle assembly and/or function due to its hybrid genetic background (de Melo Sales et al., 2024; Shamina, 2005; Shamina et al., 2003). However, since *L.s.* and *L.a.* are original inbred species, the mechanisms that underpin the potential attenuated spindle function in *Lycoris* and petunia are not likely the same. We have tried immunolocalization of microtubules using an antibody available for Arabidopsis (Lei et al., 2020), but no positive microtubule signal was detected (data not shown), which may be owing to divergence of α-tubulin proteins between species, or the tested protocol awaits further modifications. Remarkably, triad-like configuration of PMCs at tetrad stage that manifests meiotic restitution was observed both in *L.s.* and *L.a*., suggesting a potential of diploid *Lycoris* to yield unreduced gametes and thus polyploid progeny (d'Erfurth et al., 2008; De Storme and Geelen, 2011). This phenomenon is highly relevant with the natural derivation of polyploid *Lycoris* considered in an ecophysiological perspective or on a large evolutionary time scale (Ramsey and Schemske, 1998). Examination of spindle and phragmoplast structures during meiosis II should be performed in future studies to decipher the cytological mechanism of triad formation in *Lycoris*, which may favor the induction of reduced gametes by genetic manipulation tools and/or application of exogenous treatment of environmental stimulus in polyploid *Lycoris* breeding programs (Fu et al., 2024; Wang et al., 2017; Zhao et al., 2023; Zhou et al., 2022).

Abundant meiotic recombination events determine genetic diversity and is crucially important for breeding program (Choi, 2017). Breaking CO limitation and modifying CO distribution at specific chromosome regions including CO cold spots have been a particular interest by researchers and breeders (Mieulet et al., 2018; Taagen et al., 2020). However, despite its significance for parental germline selection through hybridization breeding, the CO rate and distribution in *Lycoris* remain largely unknown. In this study, by quantifying the number of HEI10 foci on the pachytene and diakinesis chromosomes in *L.s.* and *L.a.*, we report that there is no significant difference in levels of type I-class CO between these two diploid *Lycoris* species. However, the similarity of CO rates in *L.s.* and *L.a.* does not necessarily reflect a general situation in a broader range of *Lycoris* species (Bauer et al., 2013; Sidhu et al., 2015; Ziolkowski et al., 2017). Similarity and difference of CO rate between closely-related species are multifacetedly controlled which involve divergence and/or conservation of molecular factors, environmental conditions and evolutionary adaption (Dumont, 2020; Smukowski and Noor, 2011). Evaluation of CO rate in other *Lycoris* species await further studies. Notably, *L.s.* and *L.a.* showed an average number of 3.0 and 4.0 COs per bivalent, respectively, which are higher than the values (approximate 1.0-2.0 COs per bivalent) generally detected in other plant species (Desjardins et al., 2022; Li et al., 2018; Sidhu et al., 2015; Steckenborn et al., 2023; Zhao et al., 2024). It should be noted that these values were calculated only based on the foci quantification of HEI10 proteins, which represent the type I COs that possibly occupy approximately 85-95% of total CO number in diploid *Lycoris* (Draeger et al., 2023; Higgins et al., 2004). Such a high CO rate may potentially damage meiotic genome stability by challenging CO intermediate resolution and thus segregation of homologs (Singh et al., 2023), which may explain the observed irregularities in unbalanced chromosome distribution. CO rate is positively correlated with the length of the bivalent structure and is negatively associated with the level of CO interference (or CO interference length) (Ernst et al., 2024; Hultén, 2011). We speculate that the relative higher CO rate in *Lycoris* could be at least partially owing to its large genome and chromosome size and thus weaker CO interference (Liu et al., 2021; Zonneveld et al., 2005). Moreover, it is of interests to explore whether the high CO rate feature in diploid *Lycoris* has any potential relationship with its special physiological trait, i.e., its meiosis occurs in bulbs underground, which supply the meiocytes with a relatively stable environmental condition compared with other plant species. Overall, our findings suggest that *Lycoris* may have evolved a nature of high CO rate during evolution and/or adaption to local environment conditions. This character may contribute to an abundant genetic diversity during natural evolution and speciation and a strong environmental adaption ability in *Lycoris*. Practically, a high level of CO formation increases the opportunities of breaking genetic linkages and promotes the selection of inbreds with interested phenotypes, which thus can facilitate the hybridization-based breeding programs in *Lycoris*.

In two allotriploid *Lycoris* varieties, defects in chromosome pairing and/or synapsis at pachytene stage were frequently visualized. The high levels of meiotic instabilities damage faithful chromosome segregation and formation of euploid gametes and thus cause sterility, which hinders reproduction and breeding of the allotriploid *Lycoris*. Apart from the odd number of chromosome sets, the pairing defects in allotriploid *Lycoris* could be partially caused by the difficulties in the recognition and interaction between homologs and homeologs and a subsequent interfered pairing process (Grandont et al., 2014; Lloyd and Bomblies, 2016). Considering the high levels of cellular defects in triploid *Lycoris*, meiosis manipulation-based breeding and development of triploid *Lycoris* varieties (maybe also some other polyploids) may need support from other breeding tools, for example, induction of genome duplication by colchicine treatment on young seedlings, which doubles chromosome sets and thus enables efficient pairing and/or other interactions between homologs. In further studies, fluorescent *in situ* hybridization (FISH) or chromosome painting techniques using DNA probes that can recognize and distinguish specific DNA regions and/or backgrounds between homologs and homeologs could be applied to confirm chromosome structures and interactions including pairing, CO formation and chromosome associations in polyploid *Lycoris* (Braz et al., 2021; He et al., 2018; Sun et al., 2022; Yu et al., 2024, 2022; Zhao et al., 2025). Notably, *L.c.* and *L.h.* showed different rates of PMCs with univalents, suggesting that triploid *Lycoris* varieties have different abilities in homologs sorting and/or pairing (Grandont et al., 2014). This difference is possibly owing to the different parental backgrounds and karyotypes (Quan et al., 2024; Zhang et al., 2025). Specifically, *L.c.* is a hybrid of *L. radiata* (*L.r.*, 2n = 22 = 22A) and *L.s.* (2n = 22 = 22A), having a karyotype of 3n = 33 = 33A, and *L.h.* is a hybrid of *L.r.* and *L.a.* (2n = 14 = 8m + 6T), having a karyotype of 3n = 29 = 4m + 22A + 3T (Li et al., 2022; Liu et al., 2019; Meng et al., 2018). The complex karyotype and composition of the chromosomes in *L.h.* may increase the possibility of chromosome rearrangements (e.g., non-homologous chromosome interaction and association) and lead to a higher level of defects in chromosome pairing, CO formation and chromosome segregation. Taken together, this study provides a cytological reference and insights into meiosis features in *Lycoris*, which paves a road for further studies on reproductive biology, genetics and practical breeding programs.

## Material and methods

### Plant materials and growth conditions

*Lycoris* (diploid species *L.* sp*rengeri* and *L. aurea*, and allotriploid varieties *L. chunxiaoensi* and *L. hubeiensis*) were cultivated in gardens during the growing season in Wuhan (30.52°N, 114.31°E).

### Correlation analysis of meiosis stage and the anther size

As shown in **Figure 1B**, the anthers of *Lycoris* were isolated from flower buds and were placed on a glass slide, after which meiocytes were extruded out of the anthers for staining, examination and staging under a microscope, and the length of the anthers were measured. For a single anther, its length value may be used for multiple times at different meiosis stages when meiocytes at the corresponding stages in this anther were observed.

### Cytological analysis of meiocytes and pollen grains

Orcein staining of meiocytes was performed by referring to (Fu et al., 2024). In brief, the anthers were cut to release meiotic products in a drop of 4.5% lactopropionic orcein solution followed by microscopic examination. For fluorescein diacetate (FDA) staining, mature pollen grains in anthers of *Lycoris* were released in a drop of FDA staining buffer (2 mg/mL in acetone) on a glass slide, and the fluorescence was observed after 10 min of staining. In both diploid and triploid *Lycoris*, hundreds of pollen grains were counted to calculate the rate of viable pollen.

### Preparation of chromosome spreads

Chromosome spreading was performed by referring to (Ross et al., 1996) with minor modifications. In brief, anthers at meiosis stages fixed in cold Carnoy’s fixative for at least 24 h were washed twice in distilled water and once in citrate buffer (10 mM, pH = 4.5), followed by incubation in a digestion enzyme mixture (0.3% pectolyase and 0.3% cellulase in citrate buffer) at 37°C for 3 h. Digested flower buds were subsequently washed once in distilled water, which thereafter were macerated in distilled water on a glass slide. Two rounds of 60% acetic acid were added to the slide, which was dried on a hotplate at 45°C. The slide was flooded with cold Carnoy’s fixative and then was air dried. 4’,6-diamidino-2-phenylindole (DAPI) was diluted to 5 μg/mL in Vectashield antifade mounting media.

### Immunolocalization and quantification of fluorescent foci

Immunostaining of HEI10 was performed as previously reported with minor modifications (Wang et al., 2014). In brief, the meiotic chromosomes were fixed on glass slides by performing chromosome spreading. The slides were then treated with microwave for 1–2 min in a jar with citrate buffer (10 mM, pH = 4.5) without letting the liquid boiling. The slides were immersed in 1 X PBS buffer containing 0.1% Triton X-100 for 30 min, after which were transferred to a humid box and were treated with a blocking buffer (2% BSA with 0.1% Triton X-100) for 30 min. The antibody against Arabidopsis HEI10 protein (rabbit) (Fu et al., 2022) was diluted by 1:100, and was then added to the slides, which were kept in the humid box at 4°C under dark for 24 h. The primary antibody was washed out by 1 X PBS buffer with 0.1% Tween-20 for three times (each time 10 min). The secondary antibody Goat anti-Rabbit IgG (H+L) Cross-Adsorbed Secondary Antibody Alexa Fluor 555 (Invitrogen, A32732) was diluted to 10 µg/mL, and was added to the slides which were then kept in the humid box at 25°C under dark for 2 h. After washes by 1 X PBS buffer with 0.1% Tween-20 for three times (each time 10 min), DAPI was added to the slides. Image processing and quantification of fluorescent foci were performed as previously reported (Fu et al., 2022). Briefly, images taken via the DAPI and RFP channels were merged, and the foci merged onto chromosomes were considered the specific foci to the HEI10 proteins and were counted manually using the ImageJ count tool.

### Microscopy

Fluorescence microscopy was performed using an Olympus IX83 inverted fluorescence microscope equipped with an X-Cite lamp and a Prime BSI camera. Bifluorescent images and Z-stacks were processed using Image J.

### Statistical analysis

Significance was calculated using unpaired *t*-tests or Chi-squared tests with GraphPad Prism (v.8). The significance level was set as *P* < 0.05. The number of bio-replicates or cells have been indicated in the figures or figure legends.

## Data Availability

The original contributions presented in the study are included in the article/**Supplementary Material**. Further inquiries can be directed to the corresponding author.
