# Supplementary material for: Meiosis in bulbous flower species Lycoris: dances underground
Source: Front Plant Sci. 2026 Jan 2;16:1691599. doi: 10.3389/fpls.2025.1691599 (PMC12808366; doi:10.3389/fpls.2025.1691599)
Supplement: Supplementary file 1 [file DataSheet1.docx]

**Supplemental Materials**

**Meiosis in bulbous flower species *Lycoris*: dances underground**

Ziming Ren^1,🖂^, Jingru Wang^1^, Nan Huang^1^, Huiqi Fu^2^, Bing Liu^2^, Yiping Xia^3^

^1^Department of Landscape Architecture, School of Civil Engineering and Architecture, Zhejiang Sci-Tech University, Hangzhou 310018, China.

^2^Arameiosis Lab, South-Central Minzu University, Wuhan 430074, China.

^3^Genomics and Genetic Engineering Laboratory of Ornamental Plants, Department of Horticulture, College of Agriculture and Biotechnology, Zhejiang University, Hangzhou 310058, China.

^🖂^To whom correspondence should be addressed: Z.R. ([zimingren@zju.edu.cn](mailto:zimingren@zju.edu.cn)).


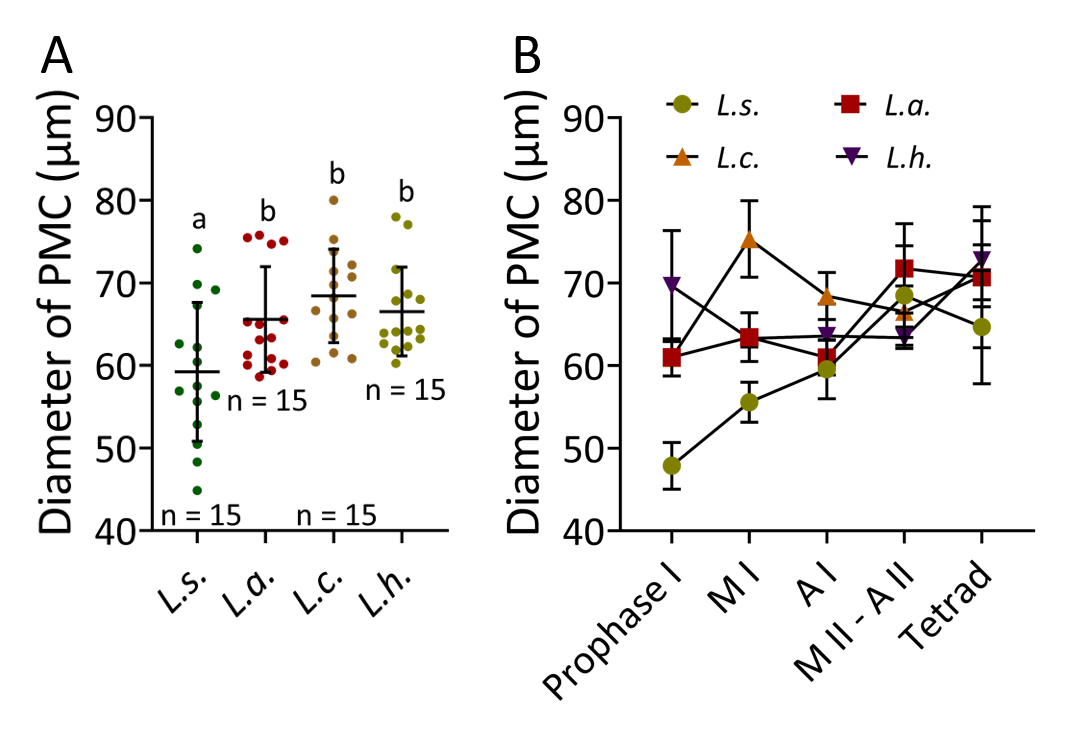


Supplementary Figure S1. Sizes of pollen mother cells (PMCs) in diploid and triploid *Lycoris*. A, Graph showing the average size of the PMCs in *L.s.*, *L.a.*, *L.c.* and *L.h.*. Significance levels were determined based on the unpaired *t* tests; different lower letters indicate *P* < 0.05; n indicates the number of analyzed PMCs. B, Graph showing the average sizes of the PMCs at prophase I, metaphase I, anaphase I, metaphase II to anaphase II and tetrad stages, respectively, in *L.s.*, *L.a.*, *L.c.* and *L.h.*. For each meiotic stage, diameters of three PMCs were calculated.


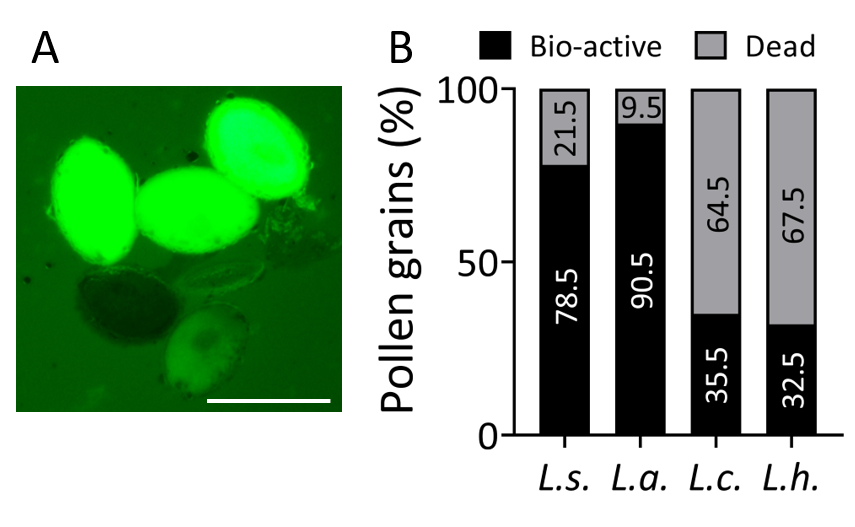


Supplementary Figure S2. Pollen viability in diploid and allotriploid *Lycoris*. A, FDA-stained pollen grains showing or not showing green fluorescence, which indicate bio-active and dead pollen grains, respectively. Scale bar = 100 μm. B, Graph showing the rates of pollen grains in *L. sprengeri*, *L. aurea*, *L. chunxiaoensis* and *L. hupehensis* showing or not showing green fluorescence. The numbers indicate the rates of the corresponding phenotypes.


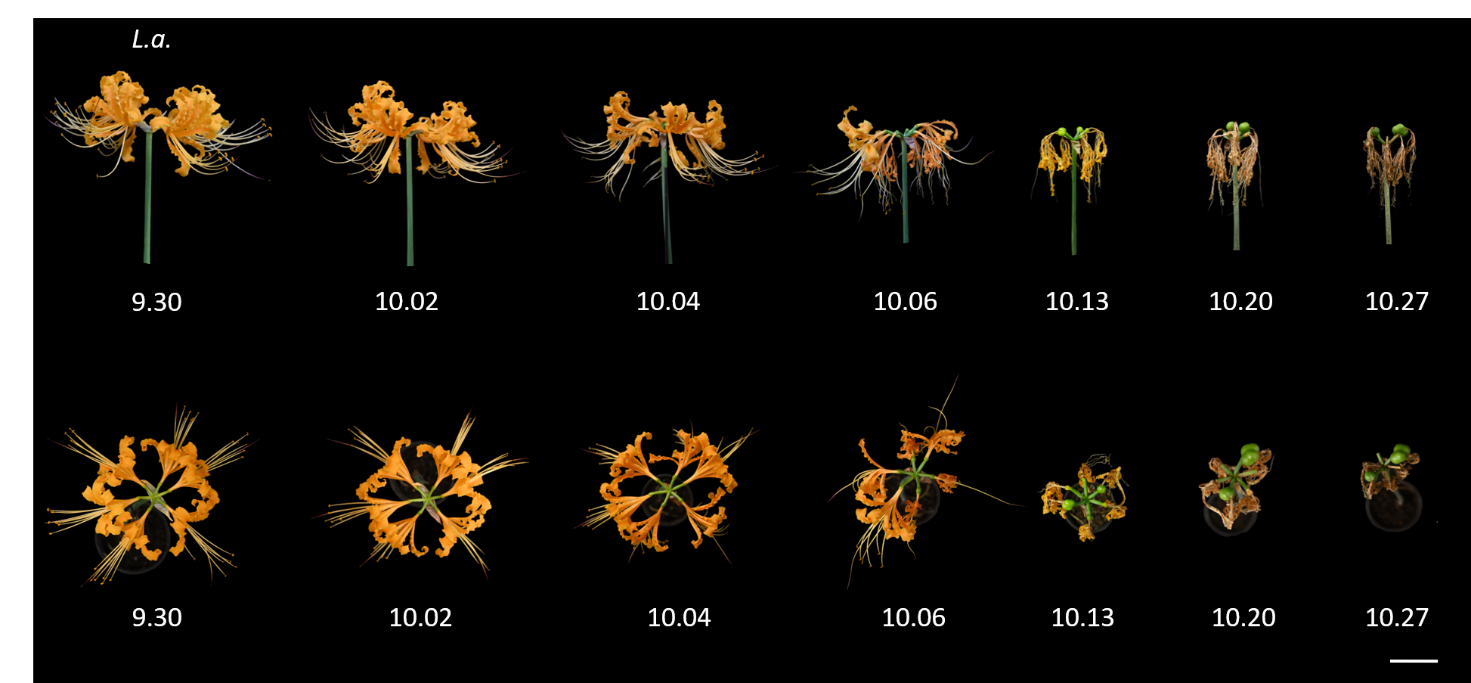


Supplementary Figure S3. Development and seed setting in *L. aurea*. Scale bar = 5 cm.


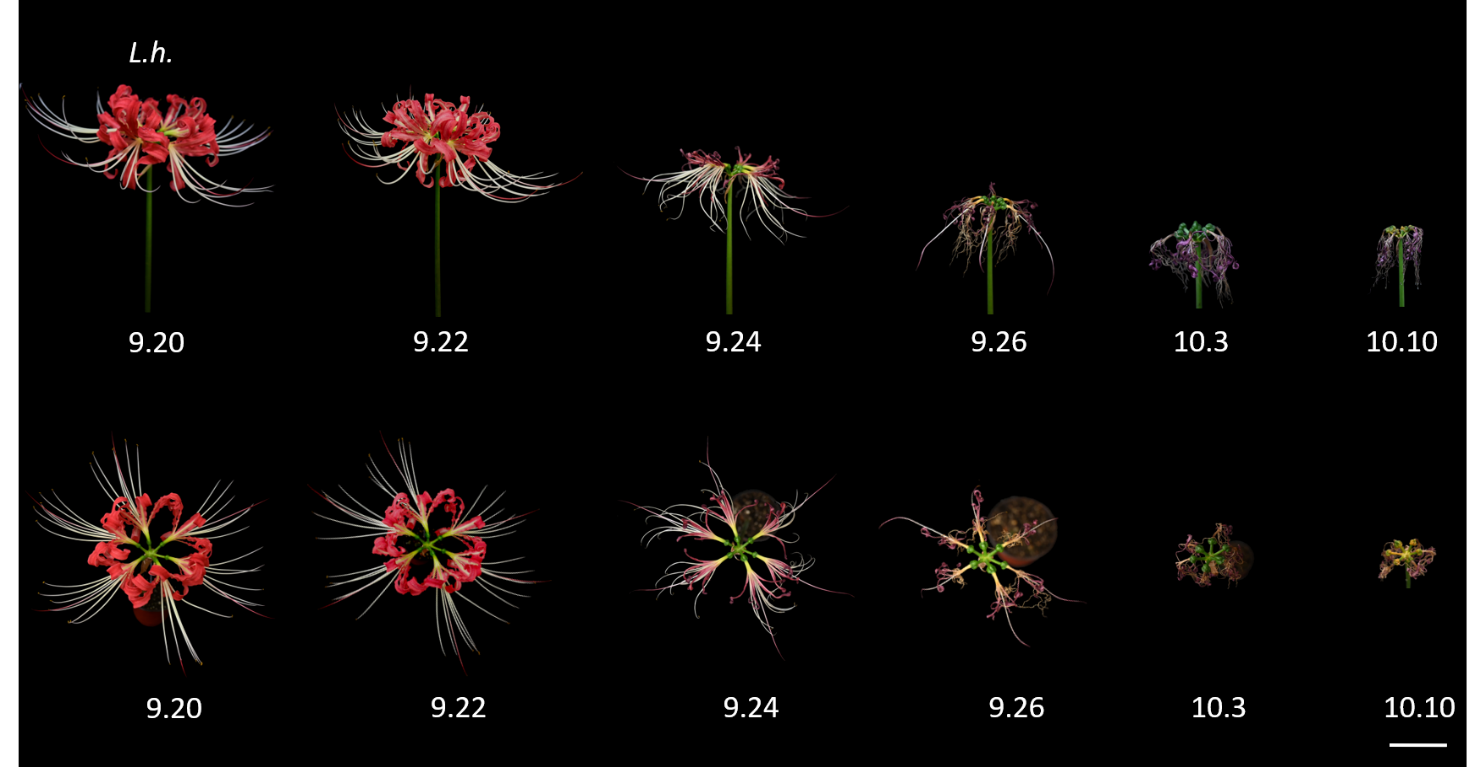


Supplementary Figure S4. Development and seed setting in *L. hubeiensis*. Scale bar = 5 cm.
